# Evaluation of Dexmedetomidine-Associated Bradycardia and Related Drug–Drug Interactions Using Electronic Health Record (EHR) and miRNA Target Analysis

**DOI:** 10.3390/cimb47121028

**Published:** 2025-12-10

**Authors:** Xinran Zhu, Suguna Aishwarya Kuppa, Robert Morris, Lan Bui, Xiaoming Liu, Angela Hill, Feng Cheng

**Affiliations:** 1 Department of Pharmaceutical Science, Taneja College of Pharmacy, University of South Florida, Tampa, FL 33612, USA; xinranzhu@usf.edu (X.Z.); skuppa@usf.edu (S.A.K.); rpmj528@gmail.com (R.M.); 2 Department of Mathematics & Statistics, College of Art and Science, University of South Florida, Tampa, FL 33620, USA; buil@usf.edu; 3 Genomics Center, College of Public Health, University of South Florida, Tampa, FL 33612, USA; xiaomingliu@usf.edu; 4 Department of Pharmacotherapeutics & Clinical Research, Taneja College of Pharmacy, University of South Florida, Tampa, FL 33612, USA; ahill2@usf.edu

**Keywords:** dexmedetomidine, bradycardia, EHR, miRNA, DDI, pharmacovigilance, ADE

## Abstract

Dexmedetomidine is a commonly used sedative because it has minimal adverse effects on respiratory function. Nevertheless, its cardiovascular safety profile, particularly bradycardia risk and drug–drug interactions (DDIs), remains incompletely understood. Additionally, current studies, including our previous analysis using the FDA adverse event reporting system (FAERS), hold several limitations. In this study, the electronic health record (EHR) platform TriNetX was utilized for pharmacovigilance analyses of dexmedetomidine. The significantly elevated incidence of bradycardia in dexmedetomidine-treated patients was demonstrated compared to other prevalent anesthetics. Age-stratified analyses revealed pronounced susceptibility in geriatric patients, while a slightly increased susceptibility in male patients was observed. In addition, elevated DDIs of dexmedetomidine with risperidone and albuterol were identified using disproportionality analysis with propensity score matching. Finally, to investigate molecular mechanisms of dexmedetomidine-associated bradycardia, analyses were conducted on a public microarray dataset, and nine differentially expressed miRNAs were identified following dexmedetomidine administration. Gene Ontology (GO) analysis of target genes of all five up-regulated miRNAs revealed rhythmic process and muscle tissue development as potential explanations. Notably, the target genes of the up-regulated miRNAs miR-26a-5p and miR-30c-5p were significantly enriched in GO terms associated with bradycardia. Together, this study identified bradycardia as a significant adverse drug event (ADE) of dexmedetomidine administration, observed possible clinically meaningful DDIs with dexmedetomidine, demonstrated a greater risk in elderly patients, and provided transcriptomic evidence that miRNA-mediated pathway dysregulation may contribute to dexmedetomidine-associated bradycardia.

## 1. Introduction

Dexmedetomidine, commercially available under the brand name Precedex, is a potent and highly selective α2-adrenergic receptor agonist [1,2]. Its pharmacological effects are mediated through suppression of sympathetic nervous system activity and inhibition of norepinephrine release, leading to a reduction in blood pressure and the induction of sedation [3]. Dexmedetomidine also demonstrates analgesic effects. Dexmedetomidine produces its pharmacological effects primarily through potent and highly selective activation of the α2-adrenergic receptor, with particularly strong affinity for the α2A subtype. Centrally, dexmedetomidine acts on α2-adrenergic receptors located in the locus coeruleus, resulting in suppression of noradrenergic neuronal firing and a corresponding reduction in sympathetic outflow [4]. This mechanism accounts for its distinctive sedative profile [5].

Clinically, dexmedetomidine is primarily indicated for procedural sedation [6], and is also utilized as an adjunct in the management of delirium, opioid-sparing strategies, and neuroprotection [7,8,9,10,11,12,13]. For intensive care unit (ICU) sedation, the dosages are 1 mcg/kg IV over 10 min and 0.2–0.7 mcg/kg/h titration for maintenance. For procedural sedation, the dosages are 1 mcg/kg IV over 10 min and 0.6 mcg/kg/h titration for maintenance [14,15]. Since its approval by the U.S. Food and Drug Administration (FDA) in 1999 [5], the use of dexmedetomidine has been increasing substantially, owing to its favorable sedation profile characterized by easy arousability and minimal impact on spontaneous respiration [16,17]. Dexmedetomidine exhibits approximately 1620:1 selectivity for α2-adrenergic receptors over the α1 isoform, thereby minimizing adverse respiratory effects [18,19]. In addition, dexmedetomidine pretreatment has exerted cardioprotective effects against myocardial ischemia/reperfusion by activating the PI3K/Akt signaling pathway [20,21]. Retrospective studies conducted in the United States have documented a marked increase in its utilization, with ventilated adult ICU use reaching 8.6% in 2020 [22], and pediatric ICU use increasing from 6.2% to 38.2% from 2007 to 2013 [23]. Moreover, dexmedetomidine is frequently co-administered with other sedatives, anesthetic agents to help maintain hemodynamic stability by decreasing the dose of other agents, and adjunctive medications [24,25,26], which amplifies the need for a comprehensive safety profile and careful evaluation of potential drug–drug interactions (DDIs) to ensure patient safety during polypharmacy sedation strategies.

Despite its therapeutic advantages and prevalence, dexmedetomidine use is restricted by its frequently reported adverse drug events (ADEs) and unclear safety profile. Notably, bradycardia is the most common side effect of dexmedetomidine across randomized trials and meta-analyses [22,27,28,29,30,31,32,33,34]. Bradycardia, defined as a resting rate below 60 bpm in adults (and variably adjusted by age in pediatric patients), is the most encountered bradyarrhythmia in anesthesia and sedation contexts [35]. While often asymptomatic, bradycardia could escalate to significant hemodynamic compromise, potentially culminating in hypotension, syncope, cardiac arrest, or even death. A review of nearly 4000 anesthesia-related ADEs has reported that bradycardia was implicated in 25% of cases leading to cardiac arrest [36]. A clinical review found that bradycardia occurred in approximately 6.5 per 1000 procedural sedation settings [37]. Regarding dexmedetomidine, pediatric studies among mechanically ventilated children have reported a significantly elevated risk of dexmedetomidine-associated bradycardia (Odds ratio (OR) = 6.14, 95% CI: 2.20–17.12) compared to other sedatives [29]. Another meta-analysis has demonstrated an increased incidence of bradycardia (OR = 5.13, 95% CI: 0.96–27.47) after dexmedetomidine administration in post anesthesia care unit (PACU) patients [38]. However, most of the current systematic reviews and meta-analyses of dexmedetomidine-associated bradycardia are constrained by several methodological and contextual limitations that complicate inference and lead to divergent conclusions. First, much of the evidence arises from single-region or single-center cohorts and narrowly defined clinical settings (e.g., cardiac surgery, sedation in ICU, or pediatric imaging), limiting external generalizability [33,34,39]. Second, modest patient numbers or event counts reduce the statistical power to detect ADEs and contribute to imprecision [40,41]. For example, several studies reported large deviations with sometimes insignificance for the OR of dexmedetomidine-associated bradycardia [38]. Third, for real-world observational studies, heterogeneity of populations in exposed and control groups for comparison could lead to bias and distort the relationship between exposure and outcomes. Lastly, the lack of a DDIs’ safety profile of dexmedetomidine in current studies underscores the need for a systematic assessment of the co-administration of dexmedetomidine with other agents on cardiovascular effects.

Our previous work, entitled “The Association Between Dexmedetomidine and Bradycardia: An Analysis of FDA Adverse Event Reporting System (FAERS) Data and Transcriptomic Profiles”, employed a multidisciplinary approach that integrated pharmacovigilance and transcriptomic analyses [30]. Bradycardia was demonstrated as the most frequent ADE associated with dexmedetomidine, followed by hypotension and cardiac arrest, by association rule mining and disproportionality analysis of FAERS reports. In addition, transcriptomic analysis using a public RNA-seq dataset revealed eight genes related to cardiac muscle contraction that were significantly downregulated in mouse cardiac cells exposed to dexmedetomidine. Potential DDIs were also identified, notably with Lactated Ringer’s Solution, bupivacaine, risperidone, and albuterol [30].

Despite these findings, this study was limited by several factors. First, the pharmacovigilance analysis relied on a relatively small number of FAERS reports (*n* = 1611), which restricted the statistical power. Second, the lack of propensity score matching raised concerns about residual confounding and possible bias in the observed associations. These limitations motivated the present study, which utilizes electronic health record (EHR) data to validate and expand upon our previous findings. EHR refers to a digital version of a patient’s medical chart maintained by hospitals or clinics, usually containing real-time and longitudinal patient data, including demographics, diagnoses, medications, lab results, and clinical notes. The rich clinical context allows tracking of drug exposure and outcomes over time and supports real-world evidence-based analyses [42]. Compared to FAERS, EHR offers a vastly larger sample size (on the order of 100 million records) enabling more robust and comprehensive analyses. The incorporation of propensity score matching minimizes bias by eliminating confounding factors. Moreover, the scope of transcriptomic investigation was broadened by introducing miRNA-based studies, which complement our prior mRNA-level analyses.

Therefore, this study aimed to validate the association between dexmedetomidine and bradycardia using large-scale EHR data, assess the impact of demographic confounders, identify clinically meaningful DDIs, and explore potential molecular mechanisms using miRNA expression profiling. By integrating real-world evidence with transcriptomic analyses, we sought to provide a more comprehensive and mechanistically informed evaluation of the cardiovascular safety profile of dexmedetomidine.

## 2. Materials and Methods

### 2.1. EHR Data and Propensity Score Matching

EHRs were accessed and analyzed the using real-world data network TriNetX (http://www.trinetx.com/ (accessed on 15 November 2025)). US Collaborative Network of the database was selected in this study.

Cohorts were defined by the following terms: “ICD-10-CM R00.1 Bradycardia, unspecified”, “48,937 dexmedetomidine”, “8782 Propofol”, “6130 Ketamine”, “6960 Midazolam”, “35,636 risperidone”, “435 albuterol”, “J7120 Ringers lactate infusion, up to 1000 cc”, and “1815 bupivacaine” in the TriNetX databases. Bradycardia events were identified using the terms “ICD-10-CM R00.1 Bradycardia, unspecified”. In TriNetX, exposure-outcome relationships are defined using standardized time window. In this study, time window was set as “from 1 month to 1 day”.

Patient numbers, demographic data, and clinical outcomes were all accessed and compared through the TriNetX platform. For propensity score matching, cohorts were matched using propensity scores on age at index, gender (Male, Female), and race (White, Black or African American, and Asian). TriNetX applies 1:1 nearest-neighbor propensity score matching using the user-selected covariates. The platform performs matching automatically, and matched cohorts are balanced on these variables.

### 2.2. Disproportionality Analysis

For disproportionality analysis, exposed and control cohorts’ definitions were annotated specifically. Odds ratios (ORs) were calculated to quantify the disproportionality using the standard formula:

OR = (a × d)/(b × c)(1)

where

a = number of events in the exposed group,
b = number of events in the control group,
c = number of non-events in the exposed group,
d = number of non-events in the control group.

### 2.3. FAERS Data Analysis

Adverse event reports submitted through FAERS were accessed using the publicly available FAERS dashboard tool. Both generic drug terms and the most common brand names were used as input search terms (dexmedetomidine: dexmedetomidine, dexmedetomidine hydrochloride, Precedex; Propofol: Propofol, Diprivan; Ketamine: Ketamine, Ketamine hydrochloride, Ketalar; Midazolam: Midazolam, Midazolam hydrochloride, Vesed).

To avoid repeated reports, indirect reports extracted from publications were excluded from analysis. Data analysis was performed using R statistical software version 4.5.0.

### 2.4. Transcriptomic Data Analysis

A public microarray dataset from the National Center for Biotechnology Information (NCBI) Gene Expression Omnibus (GEO) database (GEO id: GSE126106) was analyzed to explore the differentially expressed miRNAs in rat hearts of control groups (saline infusion for 30 min) and dexmedetomidine groups (dexmedetomidine infusion for 30 min). Differentially expressed genes were identified using the Limma package, which computes Benjamini–Hochberg (BH)-adjusted *p*-values to control the false discovery rate (FDR). Only miRNAs with an adjusted *p*-value < 0.05 and the absolute value of fold change >2 were considered significantly differentially expressed. GO enrichment analysis was performed using the clusterProfiler package with annotations from org.Hs.eg.db. Gene symbols were converted to Entrez Gene identifiers via the bitr function, and enrichment results were calculated with the BH method for multiple testing correction. All analyses were conducted in R statistical software version 4.5.0. miRNA target prediction was performed on TargetScanHuman 8.0 (https://www.targetscan.org/vert_80/ (accessed on 15 November 2025)).

## 3. Results

### 3.1. Association Between Dexmedetomidine Administration and Increased Risk of Bradycardia

First, we identified bradycardia as a significant ADE associated with dexmedetomidine using TriNetX data. As shown in Table 1, bradycardia occurred in 10.972% of patients receiving dexmedetomidine (*n* = 3,261,825), compared to 2.257% in the control group (patients not receiving dexmedetomidine, *n* = 128,539,168). The incidence in the dexmedetomidine group was nearly four times higher than in the control group, indicating a strong association between dexmedetomidine and bradycardia. These findings are consistent with our previous analysis using FAERS data.

To determine whether the observed association between dexmedetomidine and bradycardia was due to other general anesthetics commonly used in perioperative settings rather than dexmedetomidine itself, we compared ADE profiles for dexmedetomidine with three widely used anesthetics including propofol, ketamine, and midazolam [43,44,45,46,47], using both FAERS data (Table 2) and propensity score-matched TriNetX data (Table 3).

FAERS data analysis showed that, using dexmedetomidine as the reference, all three comparators had significantly lower ORs, ranging from 0.165 (95% confidence interval (CI): 0.132–0.207) for ketamine to 0.285 (95% CI: 0.242–0.335) for propofol, with CIs excluding 1. Additionally, the rank of bradycardia among reported ADEs for these drugs was consistently lower (propofol: rank 7; ketamine: rank 32; midazolam: rank 8) compared with dexmedetomidine, for which bradycardia ranked first. These findings indicate that dexmedetomidine showed a significantly higher risk of bradycardia than the other general anesthetics.

For EHR data analysis, demographic biases between cohorts could potentially lead to confounding conclusions [48,49]. Without controlling baseline differences, associations between medications and outcomes may reflect underlying population characteristics rather than true pharmacological effects. Therefore, in our analysis, propensity score matching was applied to two cohorts to control key demographic factors, including age, gender, and race, thereby minimizing potential confounding [49]. Across all matched comparisons, using dexmedetomidine as the reference, propofol, ketamine, and midazolam exhibited ORs below 1 with 95% CIs excluding 1 (propofol: OR = 0.519, 95% CI: 0.513–0.525; ketamine: OR = 0.711, 95% CI: 0.703–0.719; midazolam: OR = 0.576, 95% CI: 0.569–0.582). These findings, consistent across FAERS pharmacovigilance data and real-world EHR analyses, demonstrate a significantly stronger association between dexmedetomidine and bradycardia compared with other general anesthetics, confirming that the observed cases are not merely attributable to anesthetic or surgical conditions but represent an intrinsic safety signal of dexmedetomidine.

### 3.2. Age-Stratified Analysis of Dexmedetomidine-Associated Bradycardia Risk

**Figure 1:**
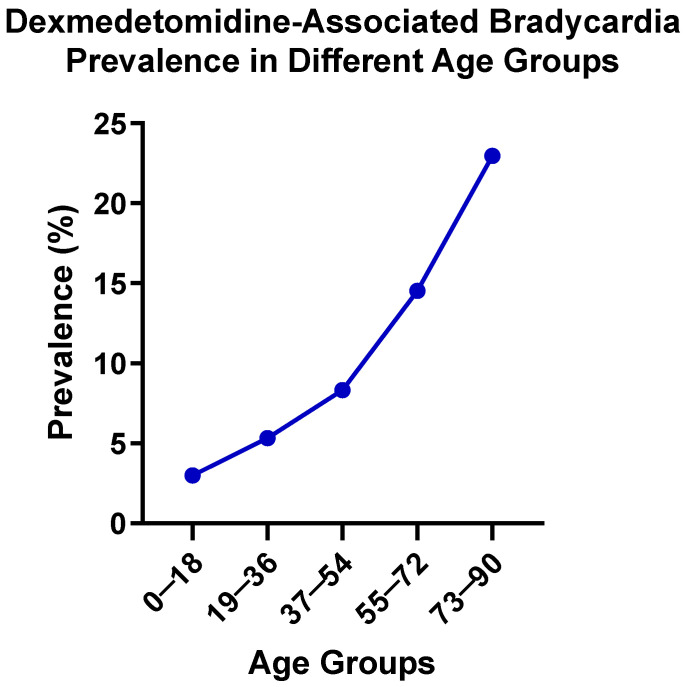
Dexmedetomidine-associated bradycardia prevalence in different age groups.

To find potential age- and gender-specific susceptibility to bradycardia in patients administered dexmedetomidine, age- and gender-stratified analyses of dexmedetomidine-associated bradycardia risk were conducted using EHR data. Figure 1 illustrates the age-stratified prevalence of dexmedetomidine-associated bradycardia across five age groups. The prevalence of bradycardia demonstrated a clear age-dependent gradient, rising steadily from 2.984% in patients aged 0–18 years to 22.976% in patients aged 73–90 years. The observed age-related trend suggests that older patients are at substantially greater risk of experiencing bradycardia, emphasizing the importance of the careful monitoring of bradycardia in elderly populations during dexmedetomidine treatment.

### 3.3. Gender-Stratified Analysis of Dexmedetomidine-Associated Bradycardia Risk

Table 4 presents the prevalence of dexmedetomidine-associated bradycardia by sex. The prevalence was slightly higher in males (12.084%) compared with females (9.840%), suggesting a modestly increased risk of bradycardia in male patients receiving dexmedetomidine. This finding highlights the potential need to consider sex as a factor when monitoring ADEs during dexmedetomidine treatment.

### 3.4. DDIs of Dexmedetomidine-Associated Bradycardia

Next, potential DDIs found in our previous study using FAERS were validated bby performing a disproportionality analysis of TriNetX EHR data. Table 5 summarizes the ORs of bradycardia in patients receiving dexmedetomidine in combination with one of four drugs (risperidone, albuterol, Lactated Ringer’s Solution, or bupivacaine) compared to those not receiving the respective co-medication, using data that were processed by propensity score matching. For each drug, ORs were calculated using the group that received dexmedetomidine without the secondary drug as reference. For risperidone, the OR for bradycardia in the dexmedetomidine plus risperidone group was 1.797 (95% CI: 1.688–1.913). A similar pattern was observed with albuterol (OR = 1.691, 95% CI: 1.664–1.719). These elevated ORs, with 95% CIs excluding 1, support the presence of DDIs between dexmedetomidine and these agents. In contrast, combinations with Lactated Ringer’s solution and bupivacaine yielded ORs of 0.863 (95% CI: 0.846–0.881) and 0.896 (95% CI: 0.882–0.910), respectively, indicating no evidence of DDIs associated with bradycardia based on TriNetX data.

### 3.5. miRNA Profiling Analyses of Rat Hearts Treated with Dexmedetomidine

**Figure 2:**
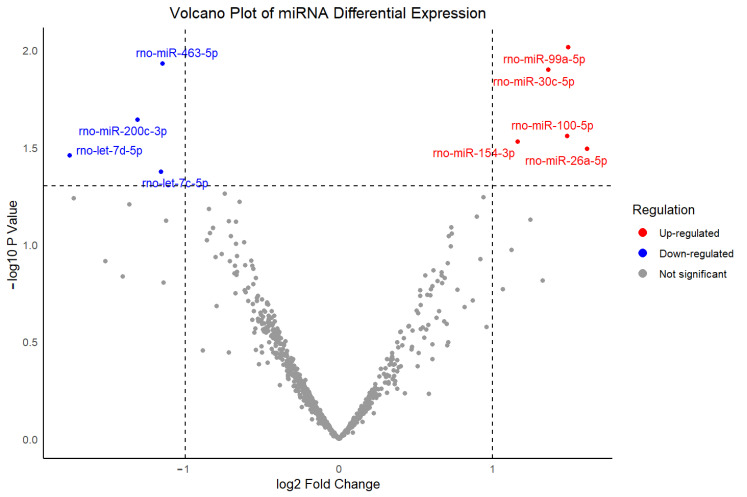
Volcano plot of differentially expressed miRNAs between dexmedetomidine treatment and control at *p*-value < 0.05 and a fold change >2.

To investigate potential molecular mechanisms underlying dexmedetomidine-associated bradycardia, transcriptomic analysis was conducted using a publicly available dataset (GEO ID: GSE126106) using the Limma package. This dataset profiled miRNA expression changes in rat hearts following dexmedetomidine administration compared to the control group using 3D-Gene Rat Oligo microarray chip 20 k V1.2.0. As is shown in Figure 2, 5 up-regulated (rno-miR-99a-5p, rno-miR-30c-5p, rno-miR-100-5p, rno-miR-154-3p, and rno-miR-26a-5p) and 4 down-regulated miRNAs (rno-miR-463-5p, rno-miR-200c-3p, rno-let-7d-5p, and rno-let-7c-5p) were identified among a total of 807 miRNAs, based on a *p*-value < 0.05 and a fold change > 2.

**Figure 3:**
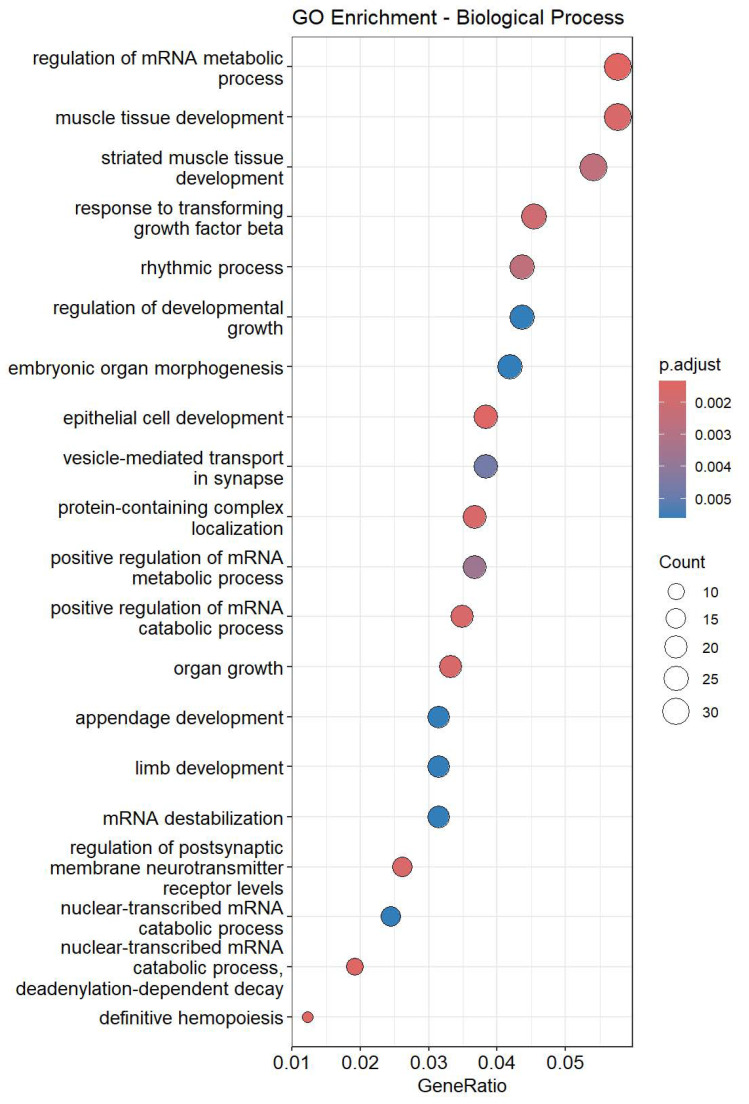
Top 20 enriched GO terms in rat hearts treated with dexmedetomidine.

These findings highlight specific miRNAs as potential mediators of the observed cardiac rhythm disturbances caused by dexmedetomidine administration. Subsequently, targeted-mRNAs of up-regulated miRNAs were predicted using Target Scan Human 8.0, followed by Gene Ontology (GO) enrichment analyses (Figure 3). GO analysis results indicated significant enrichment in processes related to rhythmic regulation, muscle tissue development, and striated muscle tissue development, all of which are closely linked to cardiac conduction and contractile function. The integration of these pathways supports a model in which dexmedetomidine-induced miRNA dysregulation contributes to altered molecular signaling, thereby leading to bradycardia.

To further identify individual miRNAs that are most strongly associated with dexmedetomidine-induced bradycardia, separate GO enrichment analyses were conducted. To systematically evaluate their potential contribution to bradycardia, a reference list of bradycardia-related GO terms was compiled first (Table S1). A bradycardia relationship score was calculated for each miRNA using the following formula:

score_sum = ∑ (−log_10_ (adjust *p*-value for bradycardia-related GO terms)) (2)

Thereby, the extent of each miRNA’s enrichment in bradycardia-relevant pathways was quantified.

Among the 5 up-regulated miRNAs, 2 miRNAs, miR-26a-5p and miR-30c-5p, demonstrated markedly higher scores for GO terms compared with others, for which the scores were zero (Table 6). Regulation of heart rate and regulation of cardiac muscle contraction were found enriched in miR-26a-5p targeted mRNAs, while cardiac muscle cell action potential, heart process, and heart contraction were found enriched in miR-30c-5p targeted mRNAs.

Together, miRNA analyses identified 9 differently expressed miRNAs under dexmedetomidine treatment. Rhythmic regulation and muscle development pathways were found to be enriched in the 5 up-regulated miRNA-targeted mRNAs. Furthermore, two miRNAs, miR-26a-5p and miR-30c-5p, were identified as candidates that are potentially involved in dexmedetomidine-associated bradycardia.

## 4. Discussion

This study employed a multidisciplinary approach integrating large-scale EHR analyses with transcriptomic profiling to evaluate bradycardia as a clinically significant ADE associated with dexmedetomidine. Stratified analyses by age and gender revealed distinct risks of dexmedetomidine-induced bradycardia, with particularly elevated risk observed among elderly patients. In addition, risperidone and albuterol were observed as being associated with higher bradycardia risk, when co-administered with dexmedetomidine. Furthermore, miRNA analyses identify possible miRNAs and their target genes that may be involved in the process by which dexmedetomidine treatment is associated with bradycardia. These findings suggest potential risks of dysregulated heart rate control associated with the use of dexmedetomidine, emphasizing the importance of careful risk assessment and clinical monitoring, particularly in geriatric patients and cases of co-administration with other medications.

The pronounced increase in bradycardia prevalence observed with advancing age among patients treated with dexmedetomidine may reflect a convergence of age-related physiological, pharmacodynamic, and pharmacokinetic changes. First, aging is characterized by reduced autonomic flexibility, including diminished baroreceptor sensitivity, decreased β-adrenergic receptor responsivity, and increased vagal predominance [50,51,52,53], which may amplify the bradycardia effect of α2-adrenergic agonists like dexmedetomidine. Second, structural and electrophysiological remodeling of the aging myocardium, including sinoatrial node fibrosis, loss of pacemaker cells, altered ion channel expression, and slowed conduction pathways [54,55,56,57], making the older heart inherently more sensitive to suppression of nodal automaticity and conduction delays induced by dexmedetomidine. Third, pharmacokinetic and pharmacodynamic changes in older patients, such as decreased hepatic clearance, increased volume of distribution, and altered receptor sensitivity [58,59,60,61], may exacerbate the systemic and cardiac effects of dexmedetomidine. Finally, older patients often harbor comorbidities, for example, conduction system diseases that blunt heart rate, and polypharmacy [62,63,64]. Both may act as potential factors for dexmedetomidine-induced bradycardia. Collectively, these mechanisms provide a plausible explanation for the age-dependent gradient of dexmedetomidine-associated bradycardia risk observed in this study. These findings emphasize the importance of precise monitoring and dose adjustment of dexmedetomidine in elderly populations to avoid cardiovascular complications. However, it should be noted that TriNetX does not provide detailed patient-level information on comorbidities, concurrent cardio-depressant medications, dexmedetomidine infusion dose, or duration. As older patients may have a higher burden of cardiac disease and may receive longer or higher-dose infusions, as well as more concomitant medications that can depress heart rate, these factors, which are not incorporated into the stratified models, may lead to the observed increase in bradycardia prevalence with increased age being a product of either residual confounding or clinical practice patterns, rather than an independent age effect. These findings are therefore exploratory and hypothesis-generating, and future studies with full patient-level EHR data will be required to validate these age effects.

The observed DDI between dexmedetomidine and risperidone may be attributed to addictive pharmacodynamic effects mediated by central autonomic modulations. As a potent α2-adrenergic receptor agonist, dexmedetomidine diminishes sympathetic tone through vagal activation and baroreflex suppression, resulting in bradycardia and hypotension [5,31]. Risperidone is an antagonist of dopamine D2 and serotonin 5-HT2A receptors, of which are associated with QT-interval prolongation and arrhythmogenic risk, including torsade de pointes and sudden cardiac death [65,66]. Consequently, co-administration of dexmedetomidine and risperidone could produce additive effects that impair both heart rhythm regulation and myocardial repolarization, increasing vulnerability to bradyarrhythmia. Furthermore, both agents exert central autonomic suppression: dexmedetomidine via inhibition of central noradrenergic pathways and risperidone via dopaminergic and serotonergic modulation, potentially exacerbating bradycardia by compounding decreases in sympathetic output. At the molecular level, dexmedetomidine has been reported to influence gene expression linked to cardiac contraction, ion channels, and neurotransmitter regulation [67,68], while animal studies suggest that risperidone could induce proteomic alterations in cardiac myocytes, affecting calcium-handling proteins and gap junction constituents critical to conduction integrity [69]. Although direct evidence of combined transcriptional or translational changes is limited, it is plausible that co-exposure could amplify disruption of ion channel function and intercellular electrical coupling, thereby promoting susceptibility to conduction delays and arrhythmias.

The DDI between dexmedetomidine and albuterol is possibly characterized by pharmacodynamic opposition and potential electrolyte-mediated modulation of conduction. Albuterol is a β_2_-agonist with the primary function of promoting bronchodilation. It can exert sympathomimetic effects, including increased heart rate and myocardial contractility [70,71], which are antithetical to the pronounced bradycardia and hypotension of dexmedetomidine. However, this “tug-of-war” between autonomic branches may disturb normal cardiac conduction and exacerbate arrhythmic risk. During bradycardic states induced by dexmedetomidine, the sudden sympathetic push from albuterol may precipitate ectopic foci or conduction disturbances. Additionally, albuterol is known to induce serum hypokalemia and occasionally prolong QT interval, particularly at high doses or with repeated administration [72,73]. Hypokalemia compromises repolarization reverse, and when overlapped with bradycardia, may amplify QT prolongation and predispose to severe ventricular arrhythmias. Thus, the co-administration of dexmedetomidine and albuterol increases the risk of bradycardia through both antagonistic autonomic influences and electrolyte imbalance that can destabilize cardiac electrophysiology.

Despite this hypothesis, the observed association between albuterol and bradycardia may reflect residual confounding and clinical context. For example, albuterol is frequently administered to severely ill patients, such as those with respiratory failure, hypoxia, or sepsis, who may have a higher baseline risk of bradycardia due to disease severity and concomitant sedatives, or procedures such as intubation. Because TriNetX does not allow adjustment for ICU status, illness severity, or detailed medication patterns, these factors cannot be fully controlled. Therefore, the albuterol signal should be viewed as an exploratory association rather than a physiological effect, and further patient-level studies will be necessary to investigate this unexpected finding. Another limitation for the explanation of DDI between albuterol and dexmedetomidine is that TriNetX does not provide patient-level timestamps for medication administration. As a result, the precise temporal relationship between exposures cannot be established. For example, we cannot determine whether albuterol was administered before, after, or concurrently with dexmedetomidine, nor can we link bradycardia onset to a specific sequence of therapies. This lack of temporal resolution limits our ability to distinguish confounding by indication, acute illness severity, or procedural timing, and therefore the DDI findings, particularly the unexpected association with albuterol, should be interpreted with caution.

The findings related to Lactated Ringer’s Solution and bupivacaine should also be interpreted with caution. Both agents are closely tied to procedural or perioperative contexts, and their use likely reflects underlying clinical scenarios rather than true pharmacologic interactions with dexmedetomidine. Consistent with this, no significant increase in bradycardia risk was detected for those combinations in the EHR analysis. However, because TriNetX does not provide detailed procedural timing or contextual information, residual confounding due to clinical setting or co-interventions cannot be excluded.

The two up-regulated miRNAs identified in this study, miR-26a-5p and miR-30c-5p, may participate in molecular processes that are involved in known pharmacodynamic actions of dexmedetomidine. GO enrichment analysis revealed that regulation of heart rate and regulation of cardiac muscle contraction were enriched in miR-26a-5p targeted genes, while cardiac muscle cell action potential, heart process, and heart contraction were enriched in miR-30c-5p targeted genes. miR-26a-5p has been implicated in myocardial hypertrophy, inflammatory signaling, and arrhythmogenesis, which are processes that directly contribute to cardiac rhythm instability [74,75,76,77,78,79]. Similarly, the miR-30 family has been linked to cardiomyocyte survival, apoptosis regulation, and tissue remodeling, which modulate electrophysiological stability, thereby influencing susceptibility to bradycardia [80,81,82,83]. Altered expression of these two miRNAs may therefore increase susceptibility to bradycardia by modifying cardiomyocyte excitability or conduction system integrity, complementing the central and peripheral adrenergic mechanisms through which dexmedetomidine directly exerts its cardiovascular effects. The identification of these two miRNAs provides a potential molecular explanation for the elevated bradycardia susceptibility induced by dexmedetomidine observed in this study.

Although the microarray dataset used in this study was derived from rat cardiac tissue, targeted mRNA prediction was performed using the TargetScanHuman 8.0 database. This approach is justified by the high evolutionary conservation of most miRNA sequences between rodents and humans [84], where many mature miRNAs share identical or nearly identical sequences and nomenclature, including miR-26a-5p and miR-30c-5p [85]. Consequently, their biological roles, particularly in fundamental processes such as cardiac rhythm regulation, muscle contraction, and electrophysiological signaling, are generally preserved across species [86]. Furthermore, TargetScanHuman 8.0 provides more comprehensive annotation and coverage of predicted targets compared with rat-specific resources, thereby enhancing the functional interpretability of enrichment analyses. Nevertheless, certain species-specific differences in target gene networks may exist and limit direct extrapolation to human physiology. Future studies using human-derived samples or experimental validation are required to substantiate these predicted interactions.

An interesting question raised by our findings is whether related α2-adrenergic agonists, such as clonidine and guanfacine, would produce similar miRNA expression profiles in cardiac tissue, which would support an α2-receptor-dependent regulation of miRNA expression. Although this is a compelling hypothesis, our analyses relied on publicly available transcriptomic data. and comparable miRNA datasets derived from cardiac tissues were unavailable. Consequently, the comparison of miRNA profiles across different α2-adrenergic agonists and the determination of whether the two miRNAs identified in this study reflect a dexmedetomidine-specific or a broader class effect cannot be achieved. Further work incorporating parallel miRNA profiling of cardiac tissue after treatment with multiple and representative α2-adrenergic agonists will be crucial to validate this hypothesis.

Propensity score matching is a statistical technique designed to reduce confounding in observational studies by balancing baseline covariates between treatment and control groups [87]. By estimating the probability of receiving a given treatment conditional on observed covariates, propensity score matching allows for the construction of matched cohorts that mimic certain characteristics of randomized controlled trials. This approach has been widely applied in pharmacoepidemiology and clinical outcomes research to strengthen causal inference and mitigate bias arising from nonrandom treatment allocation [88,89,90,91]. The controversies between studies on FAERS and EHR in this study demonstrated that demographic or clinical imbalances could lead to spurious associations, such as the DDIs between dexmedetomidine and Lactated Ringer’s Solution or bupivacaine. As all analyses in this study were conducted within the TriNetX platform, which automatically applies propensity score matching, one limitation is that the underlying algorithm used by the platform is not transparent, preventing verification or customization of the matching procedure. To address this problem, we repeated the propensity score matching process ten independent times. Figure S1 presents the results of disproportionality analyses for bradycardia upon dexmedetomidine and risperidone, comparing outcomes before and after propensity score matching. Without propensity score matching, the ORs were 1.882, while the value decreased slightly to 1.759 after propensity score matching. Across 10 repeated runs, the ORs remained highly consistent, showing only minor fluctuations between iterations. The standard error of the mean (SEM) was 0.029, indicating minimal relative variability. The reduction in ORs suggests that the observed associations found using unadjusted EHR data are possibly attributed to demographic imbalances. In addition, the repeated matching trials yielded highly consistent results, as evidenced by the low SEM, indicating that the random selection process inherent to the propensity score matching algorithm in the TriNetX platform did not materially influence outcome estimates. These findings reinforce the internal consistency of the platform’s matching implementation.

Notably, several limitations of the TriNetX platform should be acknowledged. First, all EHR analyses were conducted using aggregated data available within the TriNetX platform, which does not provide access to raw, patient-level EHRs. Therefore, it is unable to manually verify diagnoses, assess clinical context, evaluate comorbidities in detail, or examine precise temporal relationships between drug administration and bradycardia onset. Second, although propensity score matching was applied to reduce confounding factors by age, sex, and race, residual from unmeasured variables, such as concurrent medications, severity of illness, or procedural factors, may still influence the observed associations. Third, the inconsistent and variable availability of comorbidity information across participating clinical sites makes incorporating comorbidities as matching variables lead to substantial reductions in sample size, thereby undermining the objective of investigating prevalence patterns in a large real-world population. Fourth, the platform does not provide detailed information about dosing, infusion rate, or duration. Because dexmedetomidine-induced bradycardia is highly dose-dependent, the absence of dosage data limits interpretation of risk magnitude. Fifth, the EHR-based disproportionality analyses cannot establish causality, and observed DDIs should be interpreted as signals requiring further mechanistic or clinical validation. Another limitation in statistics is the potential for Type I error inflation due to multiple subgroups and DDI analyses. Age-based and sex-based cohorts, as well as several DDI evaluations, were performed to explore clinically relevant modifiers of bradycardia risk. However, these exploratory analyses were not adjusted for multiple testing. The TriNetX platform does not offer built-in multiplicity correction for stratified or matched cohort comparisons, and these results should therefore be interpreted with caution. Further studies with dedicated statistical frameworks and access to raw patient-level data will be necessary to formally correct for multiple comparisons and validate these subgroup findings. Finally, because TriNetX uses automated cohort construction and matching algorithms that are not user-modifiable, some methodological details remain unclear. For example, the platform does not provide patient-level timestamps; users must select from predefined time windows rather than define a customized time-at-risk period. The lack of temporal data limits reproducibility and causal inference and should be considered when interpreting the findings. Further studies with full patient-level EHR access or institution-specific datasets will be important to incorporate more clinical covariates and to more rigorously assess the magnitude of confounding factors. Despite these limitations, the convergence of evidence across multiple analytic approaches strengthens the reliability of our findings.

In addition, several limitations of the FAERS component of this study should be noted. As with all spontaneous reporting systems, FAERS is subject to under-reporting, and the absence of denominator data prevents estimation of true incidence rates. Reporting is influenced by stimulated reporting, publicity, severity of events, and clinician awareness, all of which introduce substantial reporting bias. Although, in this paper, efforts were made to exclude publication-derived indirect reports, duplicate submissions may still occur and cannot be completely ruled out. Additionally, FAERS entries lack detailed clinical context, such as medication timing, comorbidities, and concurrent therapies, limiting the ability to adjust for confounding. More importantly, FAERS supports only the identification of disproportionality signals and cannot establish causal relationships. These constraints emphasize the need to interpret FAERS findings cautiously and reinforce the value of validating signals using complementary real-world data, such as EHR, as performed in this study.

## 5. Conclusions

In summary, this study identified the association between bradycardia and dexmedetomidine through large-scale EHR and FAERS analyses, independent of anesthetic or surgical confounding. Age- and gender-stratified analyses revealed a clear age-dependent increase in risk and a modest elevation in males. Propensity score-matched DDI analyses further identified risperidone and albuterol as possible pharmacological interactors with dexmedetomidine-associated bradycardia, while observed associations with Lactated Ringer’s Solution and bupivacaine in FAERS were likely attributable to demographic confounding. Finally, transcriptomic profiling identified nine differentially expressed miRNAs, with miR-26a-5p and miR-30c-5p emerging as candidates mechanistically linked to cardiac rhythm regulation. Together, these findings provide clinical and molecular evidence for the bradycardia risks of dexmedetomidine, underscore the importance of demographic and DDI factors, and suggest potential molecular pathways contributing to its bradycardic effects.

## Figures and Tables

**Table 1:** Bradycardia prevalence with dexmedetomidine treatment using EHR data.

	Bradycardia	No Bradycardia	Prevalence
With dexmedetomidine	357,881	2,903,944	10.972%
Without dexmedetomidine	2,900,688	125,638,480	2.257%

**Table 2:** Disproportionality analysis of bradycardia as an ADE among five common anesthetics using FAERS data.

Drug	Bradycardia	No Bradycardia	OR ^1^	95% CI	Rank of Bradycardia in ADEs
Dexmedetomidine	206	1447			1
Propofol	720	17,768	0.285	0.242–0.335	7
Ketamine	132	5622	0.165	0.132–0.207	32
Midazolam	475	13,656	0.244	0.206–0.290	8

^1^ dexmedetomidine as the reference for calculating ORs.

**Table 3:** Disproportionality analysis of bradycardia as ADE among five common anesthetics using propensity score-matched EHR data.

Drug	Bradycardia	No Bradycardia	OR ^1^	95% CI
dexmedetomidine	85,323	3,147,492		
Propofol	44,886	3,187,929	0.519	0.513–0.525
dexmedetomidine	79,821	2,611,285		
Ketamine	57,257	2,633,849	0.711	0.703–0.719
dexmedetomidine	85,331	3,147,805		
Midazolam	49,673	3,183,463	0.576	0.569–0.582

^1^ dexmedetomidine as reference for calculating ORs in each propensity score-matched comparison.

**Table 4:** Dexmedetomidine-associated bradycardia prevalence in different gender groups.

	Prevalence
Male	12.084%
Female	9.840%

**Table 5:** Disproportionality analysis of risperidone, albuterol, Lactated Ringer’s Solution, and bupivacaine with dexmedetomidine-associated bradycardia using propensity score-matched EHR data from the TriNetX database.

	Bradycardia	No Bradycardia	OR ^1^	95% CI
risperidone				
dexmedetomidine without risperidone	1585	56,314		
risperidone without dexmedetomidine	745	58,137	0.455	0.417–0.497
dexmedetomidine with risperidone	2788	55,111	1.797	1.688–1.913
albuterol				
dexmedetomidine without albuterol	23,561	1,132,509		
albuterol without dexmedetomidine	11,872	1,362,821	0.419	0.410–0.428
dexmedetomidine with albuterol	39,280	1,116,790	1.691	1.664–1.719
Lactated Ringer’s Solution				
dexmedetomidine without Lactated Ringer’s Solution	20,982	722,393		
Lactated Ringer’s Solution without dexmedetomidine	9255	771,800	0.413	0.403–0.423
dexmedetomidine with Lactated Ringer’s Solution	18,177	725,198	0.863	0.846–0.881
bupivacaine				
dexmedetomidine without bupivacaine	33,562	1,137,509		
bupivacaine without dexmedetomidine	15,533	1,526,582	0.345	0.338–0.352
dexmedetomidine with bupivacaine	30,152	1,140,919	0.896	0.882–0.910

^1^ dexmedetomidine without Drug 2 as the reference for calculating ORs.

**Table 6:** Top 2 miRNAs with the highest bradycardia-related GO scores.

miRNA	GO ID	Description	Adjust *p*-Value	Count	Score	Number of GO Terms	Score_Sum
miR-26a-5p	GO:0002027	regulation of heart rate	0.020	12	1.694	2	3.369
	GO:0055117	regulation of cardiac muscle contraction	0.021	10	1.675		
miR-30c-5p	GO:0086001	cardiac muscle cell action potential	0.015	14	1.835	3	4.695
	GO:0003015	heart process	0.036	28	1.448		
	GO:0060047	heart contraction	0.039	27	1.412		

## Data Availability

The original contributions presented in this study are included in the article/Supplementary Material. Further inquiries can be directed to the corresponding author.

## Supplementary Materials

The following supporting information can be downloaded at: https://www.mdpi.com/article/10.3390/cimb47121028/s1.

## Abbreviations

The following abbreviations are used in this manuscript:

EHR	Electronic Health Record
DDI	Drug–Drug Interaction
OR	Odds Ratio
FDA	Food and Drug Administration
ADE	Adverse Drug Event
FAERS	FDA Adverse Event Reporting System
ICU	Intensive Care Unit
GO	Gene Ontology
PACU	Postanesthesia Care Unit
CI	Confidence Interval
GEO	Gene Expression Omnibus
5-HT2A	5-Hydroxytryptamine Receptor 2A
SEM	Standard Error of Mean
NCBI	National Center for Biotechnology Information
IV	Intravenous
BH	Benjamini–Hochberg Method
