# Supplementary material for: Evaluation of Dexmedetomidine-Associated Bradycardia and Related Drug–Drug Interactions Using Electronic Health Record (EHR) and miRNA Target Analysis
Source: Curr Issues Mol Biol. 2025 Dec 10;47(12):1028. doi: 10.3390/cimb47121028 (PMC12731329; doi:10.3390/cimb47121028)
Supplement: Supplementary file 1 [file cimb-47-01028-s001.zip › cimb-4019834-supplementary.pdf]

current issues in molecular biology

MDPI

Supplementary S1

For quantification of bradycardia contribution to identify miRNAs that are most associated with dexmedetomidine-associated bradycardia, a reference list of bradycardia-related GO terms was summarized based on literature reviews for bradycardia-related score calculation depending on separate GO enrichment analyses on each miRNA.

Table S1. Bradycardia-related GO terms.

GO ID	Description
GO:0002027	regulation of heart rate
GO:0086009	regulation of heart rate by cardiac conduction
GO:0002026	regulation of the force of heart contraction
GO:0086091	regulation of heart contraction
GO:0060047	heart contraction
GO:0003015	heart process
GO:0086001	cardiac muscle cell action potential
GO:0061337	cardiac conduction
GO:0061339	regulation of cardiac conduction
GO:0007638	mechanosensory behavior involved in regulation of heart rate
GO:0008016	regulation of heart contraction by calcium ion signaling
GO:0055116	regulation of cardiac muscle contraction by regulation of calcium
GO:0055117	regulation of cardiac muscle contraction
GO:0048738	cardiac muscle tissue development

Supplementary S2

To guarantee the consistency of TriNetX platform's inherent propensity score matching algorithm, propensity score matching processes were repeated ten independent times of disproportionality analyses between dexmedetomidine with risperidone cohort and dexmedetomidine-only cohort. Without propensity score matching, the ORs were 1.882, while the value decreased to 1.759 after propensity score matching. The ORs of ten times repeated showed minor fluctuations across iterations. The standard error of mean (SEM) is 0.029, indicating low relative variability.

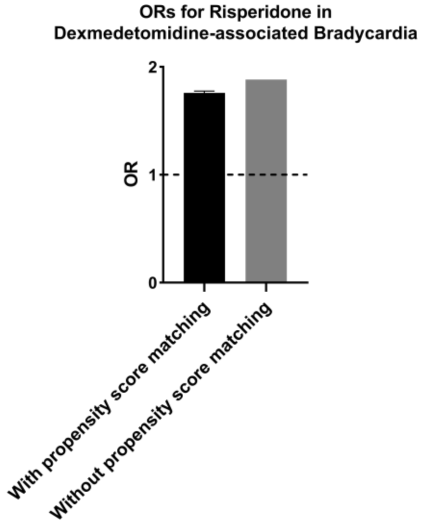

Figure S1. Comparison of ORs for risperidone with dexmedetomidine-associated bradycardia between with propensity score matching for 10 times and without propensity score matching. Without propensity score matching, the ORs were 1.882, while the value decreased to 1.759 after propensity score matching. The resulting ORs were highly consistent across 10 runs: 1.816, 1.822, 1.746, 1.837, 1.759, 1.676, 1.741, 1.686, 1.765, and 1.740 (over OR range: 1.676-1.837).
